# Genetically targeted 3D visualisation of *Drosophila* neurons under Electron Microscopy and X-Ray Microscopy using miniSOG

**DOI:** 10.1038/srep38863

**Published:** 2016-12-13

**Authors:** Julian Ng, Alyssa Browning, Lorenz Lechner, Masako Terada, Gillian Howard, Gregory S. X. E. Jefferis

**Affiliations:** 1 Department of Zoology, Downing Street, Cambridge, CB2 3EJ, United Kingdom; 2 Neurobiology Division, MRC Laboratory of Molecular Biology, Francis Crick Avenue, CB2 0QH, United Kingdom; 3 Carl Zeiss X-ray Microscopy Inc., 4385 Hopyard Rd., Suite 100, Pleasanton, CA 94588, USA; 4 Cell Biology Division, MRC Laboratory of Molecular Biology, Francis Crick Avenue, CB2 0QH, United Kingdom

## Abstract

Large dimension, high-resolution imaging is important for neural circuit visualisation as neurons have both long- and short-range patterns: from axons and dendrites to the numerous synapses at terminal endings. Electron Microscopy (EM) is the favoured approach for synaptic resolution imaging but how such structures can be segmented from high-density images within large volume datasets remains challenging. Fluorescent probes are widely used to localise synapses, identify cell-types and in tracing studies. The equivalent EM approach would benefit visualising such labelled structures from within sub-cellular, cellular, tissue and neuroanatomical contexts. Here we developed genetically-encoded, electron-dense markers using miniSOG. We demonstrate their ability in 1) labelling cellular sub-compartments of genetically-targeted neurons, 2) generating contrast under different EM modalities, and 3) segmenting labelled structures from EM volumes using computer-assisted strategies. We also tested non-destructive X-ray imaging on whole *Drosophila* brains to evaluate contrast staining. This enabled us to target specific regions for EM volume acquisition.

The ability to identify individual neurons based on their molecular, physiological and connective properties is a fundamental goal in neuroscience. A major part of this effort comes from imaging neurons, from the tracing of intricately branched axons and dendrites that span 10–100s of microns, to the identification of thin neurite endings and synaptic sites that are 10–100s of nanometre in diameter. Identifying all the patterns a single neuron makes reveals not only where the synaptic connections are and what the partner neurons may be, it also gauges its contribution to the functional network it resides in. This requires a volume-imaging approach that not only encompasses millimetre-micron wide dimensions but that also features the appropriate nanometre-scale resolutions. From a functional perspective, it is also crucial that particular neuron types can be identified based on specific molecular, morphological or physiological characteristics. For experimental paradigms, it would also be ideal if they can be easily identified between image datasets for comparative analyses. In this respect, given the complex connectivity patterns within a 3D brain environment, the specific identification and tracing of individual highly interconnected neurons from highly heterogenous neuronal populations remains a very challenging task^1^.

Recent EM techniques are well suited for large volume and high-resolution imaging, capable of capturing every axon and dendrite, synaptic and sub-synaptic feature^2^,^3^. Various volume EM techniques are currently available^2^,^3^,^4^,^5^,^6^. Serial sectioning followed by transmission electron microscopy (ssTEM) was the key method used to map the *C. elegans* nervous system, and was subsequently used in *Drosophila* and mouse^7^,^8^,^9^. Obtaining large numbers of manually generated, serial aligned and registered images from 100–1000s of sections is technical demanding^10^,^11^,^12^,^13^,^14^,^15^. Two recent imaging techniques, using serial block face scanning electron microscopy (SBF-SEM or SBEM) and focus ion beam scanning electron microscopy (FIBSEM), offer good alternatives as tissue blocks undergo automated cycles of image acquisition and sectioning that are typically well aligned^3^,^5^.

One recurrent challenge lies in image segmentation (here also termed as reconstruction)^2^,^16^. Dense reconstruction strategies involve tracing all neurites and synaptic features from the image volume, followed by feature and identity annotations^9^,^17^,^18^,^19^. Alternatively, sparse mapping strategies reduce the analysis to fewer neurons of interest, by choosing the most relevant features from the image volume for further analysis^8^,^20^,^21^,^22^. In both cases, these manual tasks are time-consuming and error-prone, and tracing needs to be repeated and expert-supervised to generate a consensus^2^,^23^,^24^,^25^. Novel computational approaches are also being deployed^26^,^27^,^28^,^29^,^30^. Nonetheless, these methods also have substantial error rates that preclude fully automated global solutions^2^. In practice, while no single procedure is perfect, multiple approaches can work in a complementary manner^2^,^17^,^24^,^30^,^31^.

EM-visible protein tags that generate electron-dense contrast can help in this effort. Acting as guides, molecularly defined neurons, synaptic sites or targets of choice can be highlighted to map circuits of interest. Such probes include enzyme peroxidases (such as HRP or APEX)^32^,^33^,^34^,^35^, photo-sensitiser proteins (such as miniSOG)^36^, and photosensitive dyes that associate with proteins^37^, or short peptides, such as ReASH^38^. These provide genetic solutions where cells can express these probes *in situ*, which are then processed for EM contrast. To date, while there have been some success, further improvements must be made to extend their targeting capabilities, labelling properties and contrast detection on the neuronal ultrastructure^32^,^33^,^36^,^39^.

Given the existing challenges in visualising the nervous system, we explored ways by which genetically encoded electron-dense probes can be used to visualise labelled neurons under various volume EM modalities. As EM projects are typically time and labour-intensive we investigated the conditions for optimal labelling, contrast staining, and sample preparation of fly brains. This was also motivated from personal experience: we find that image quality tends to be variable in terms of ultrastructure and contrast staining. Selecting specimens for EM imaging is somewhat error-prone. Without prior knowledge, sub-optimal samples are often used, resulting in wasted efforts. We investigated micro-Computed Tomography (micro-CT, also known as X-ray microscopy or XRM) as a means to assess specimen quality. While biological micro-CT/XRM is typically associated with low contrast detection in soft tissues, heavy metal and osmium-based contrast can be detected, deriving useful cell biological and neuroanatomical information^40^,^41^,^42^,^43^,^44^. With recent improvements and the availability of laboratory-based scanners, biological materials can be routinely scanned, yielding sub-micron resolutions^43^,^44^. Knowing which area to acquire EM images from is paramount as the field dimension is typically restricted, especially for TEM and FIBSEM, to about 10–20 µm^3^. This cannot be judged easily by visual inspection of the sample alone. We found that XRM could be used for this purpose. Given that this is a non-destructive process, this allows for subsequent targeted EM image acquisition.

While several electron-dense probe types are available, we focused on miniSOG^36^ and for proof-of-principle studies, labelled olfactory neurons in the *Drosophila* adult brain. We find that miniSOG labels can be targeted to distinct neuronal sub-compartments, such as membranes, cytosol, synapses and mitochondria. Through modifications in osmium and heavy metal staining, contrast introduced by the genetic label and on membrane boundaries, organelles and synaptic features can be further enhanced. The miniSOG label can be detected under TEM, SBEM or FIBSEM modalities. We explored the use of computer-based strategies to segment labelled structures throughout the volume and reveal some of the cellular and neuronal features that can be identified using these methods. Given the compatibility in contrast detection, we explored a new imaging technique, micro-computed tomography (micro-CT or X-Ray Microscopy/XRM). This allowed us to perform non-destructive X-ray imaging on intact brain samples to screen for optimally stained specimens. At submicron resolutions, macro- and micro-scale features, such as neuropil regions, axon tracts and individual cell somata could also be distinguished from these scans. This was followed with targeted volume EM acquisition to investigate features of interest at nanometre-scale resolutions.

## Results

### Design, expression and label detection of miniSOG probes

In our EM labelling strategy, we wanted to develop protein-based probes that can be selectively expressed in discrete neurons, such as through the UAS-GAL4 binary expression system^45^. The probe should also be flexible enough to label any localised region within the cell. As we aim for probe detection under electron-dense detection modality, the contrasting procedures (probe expression, localised targeting and contrast staining) must also be sufficiently robust and discernable over the surrounding cellular ultrastructure, throughout the image volume. The surrounding region should also be sufficiently contrasted for features of interest, such as synapses, organelles and the plasma membrane.

**Figure 1:**
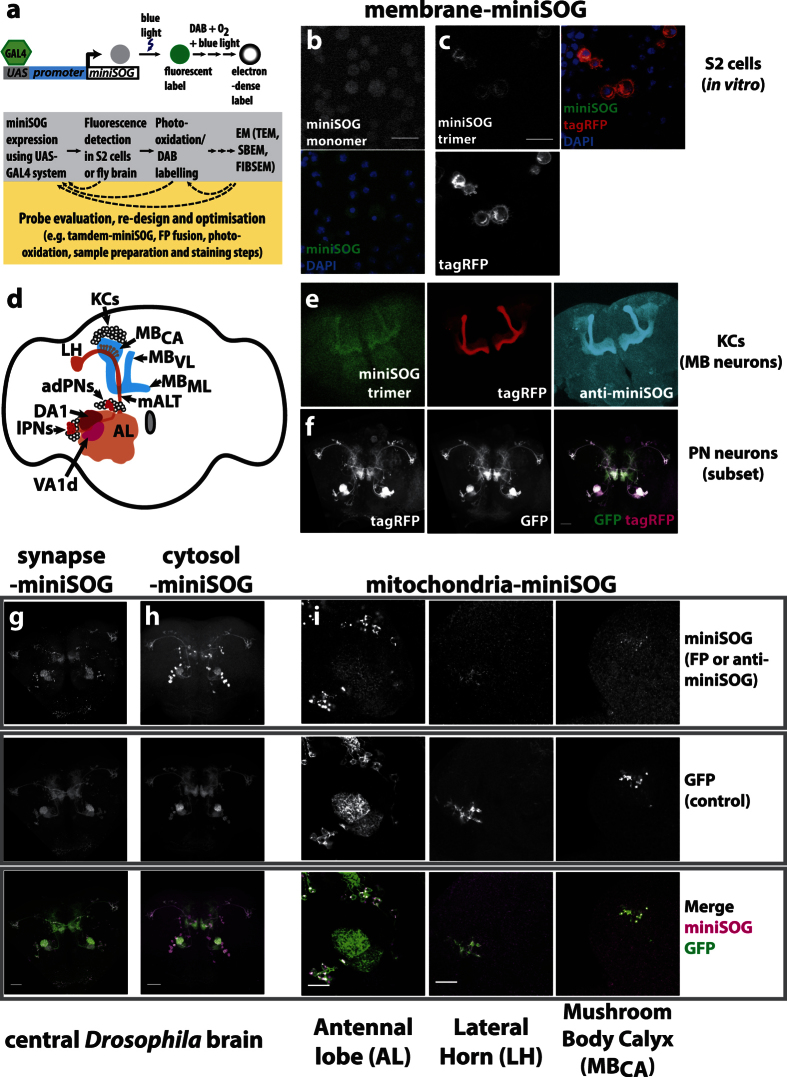
Design, expression and detection of miniSOG under fluorescence microscopy. (**a**) A design summary using miniSOG for EM-based labelling in *Drosophila.* Membrane-targeted miniSOG (membrane-miniSOG) probes were expressed either as a monomer (**b**), or trimer (**c**) in S2 cells. Fluorescence images to miniSOG, RFP or nuclear DAPI are shown. miniSOG fluorescence imaging resulted in higher backgrounds. Due to photobleaching, it was not possible to gather enough signals to detect monomeric miniSOG. Scale bars: 20 µm. (**d**) Schematic of MB neurons (also known as Kenyon cells or KCs) and a subset of projection neurons (PNs) in the *Drosophila* adult brain. From anterior dorsal (ad) or lateral (l) cell body locations, most PNs dendrites densely innervate single glomerular locations. Projecting posteriorly along the medial AL Tract (mALT) to dorsolateral locations, PN axons have long-range projections that terminate at the mushroom body calyx (MB_CA_) and lateral horn (LH)^50^. At the MB_CA_, PN axon terminals connect with KC dendrites^53^,^54^. From dorsal-posterior locations, KCs extend axon anteriorly and bifurcate to form the vertical lobe (MB_VL_) and medial lobe (MB_ML_). The UAS-GAL4 system^45^ was used to express miniSOG products in these neurons. The OK107-GAL4 labels KCs^66^. Mz19-GAL4 expression labels PN subsets, which innervated glomeruli DA1, VA1d, DC3 within the AL^67^. DC3 is not visible from the AL surface. Although these are bilaterally specified, only the left hemisphere is illustrated. (**e**) Membrane-miniSOG expression in KCs, visualised by miniSOG or tagRFP fluorescence, or by immunostaining. Despite high, non-specific background, immunolabels correlated well with miniSOG and RFP. (**f**) Membrane-miniSOG expression in PN subsets, using Mz-GAL4. As a control, a membrane-GFP reporter was co-expressed to determine the extent of overlapping projection patterns with miniSOG. Synapse (**g**), cytosol (**h**) and mitochondria (**i**) targeted miniSOG expression in PN cells, verified by fluorescence microscopy. miniSOG patterns were visualised by miniSOG immunostaining, (**h**,**i**) or through the fluorescent mKate2 fusion protein (**g**). This choice depended on the designed probe and expression levels detected. To compare overlapping expression patterns, these brains also co-expressed UAS membrane-bound GFP. Images were acquired from the brain region indicated. Scale bars: 40 µm.

We cloned miniSOG DNA into *Drosophila* UAS expression vectors that are controllable using GAL4 drivers and initially analysed these probes in cultured *Drosophila* S2 cells under fluorescence microscopy (Fig. 1a–c; Methods). We found that miniSOG fluorescence photobleached quickly under wide-field fluorescence illumination. To circumvent this, bright, photostable FPs (tagRFP or mKate2) fusions were incorporated. Our aim in probe design was to ensure miniSOG labels can be detected using fluorescence microscopy and that its expression did not cause morphological artefacts in the neurons they were expressed in. Several probes were tested in this way, targeting miniSOG to the plasma membrane, synapse, cytosol or mitochondria. Using the *Drosophila* UAS-GAL4 system^45^, their expression in olfactory neurons in the adult brain were monitored by confocal microscopy detecting either for miniSOG or FP fluorescence or by immunostainings (Fig. 1e–i). Based on light-level analysis, we verified that their expression did not cause any gross axonal or dendritic targeting defects.

As previously reported, miniSOG labelling is based on photo-oxidation^36^ where blue light illumination is used to oxidise the substrate, Diaminobenzidine (DAB). This results in a brownish, osmophillic stain, visible in brain regions where miniSOG was expressed (Supplementary Figure 1), serving as a useful indicator of the photo-conversion process. In our tests, specimens that expressed multi-copy versions of miniSOG, either as tandem motifs within a single fusion protein or multicopy transgenes showed stronger DAB labelling. Hence, the use of tandem motifs and multicopy transgenes helps to promote DAB labelling. However, careful considerations must also be taken to determine how much DAB labelling is sufficient for EM-level visualisation and ultrastructure preservation (see Discussion).

### Effect of contrasting agents on miniSOG-DAB labelling under EM

**Figure 2:**
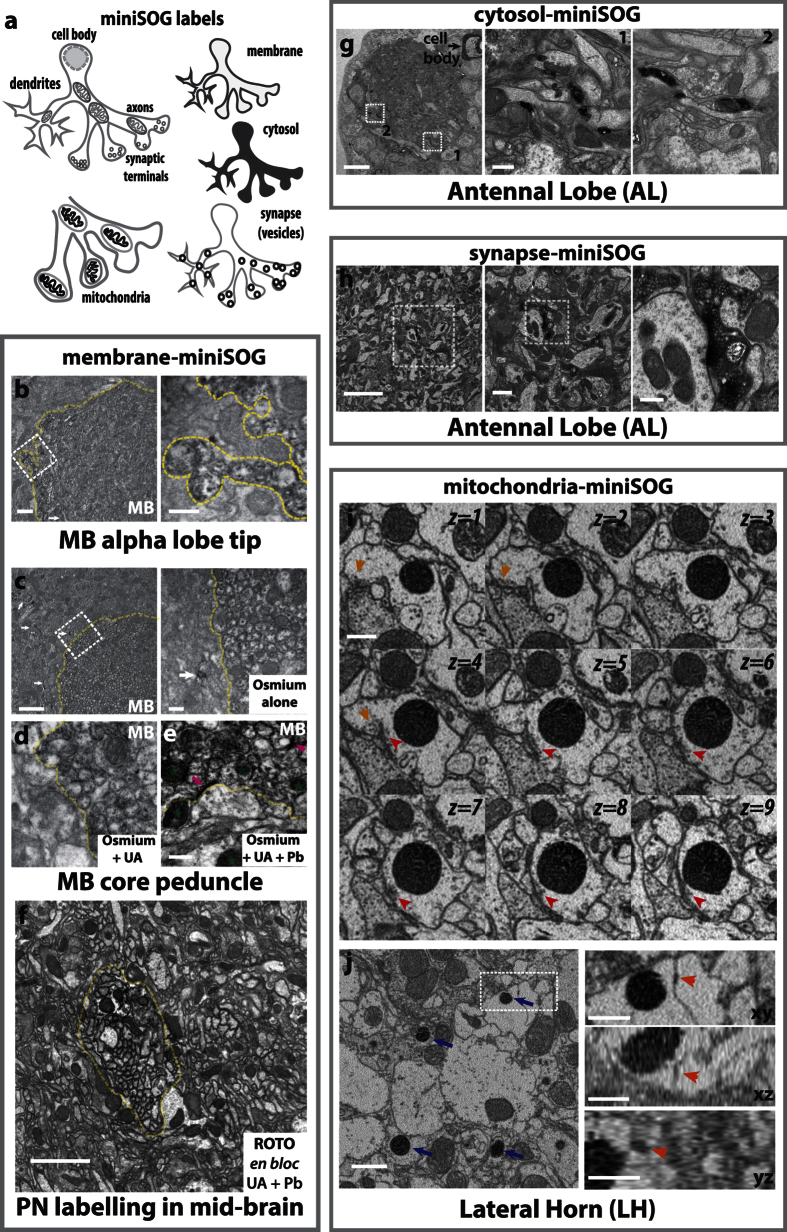
miniSOG targeting and EM contrast detection. (**a**) Summary of miniSOG probes used to label neuronal sub-compartments. (**b,c**) TEM micrographs of membrane-miniSOG labelled KCs. Higher magnifications are shown on the right. miniSOG labelling is evident on membranes and proximal cytoplasm. Dashed yellow lines: MB neuropil border. Using OK107-GAL4, some non-KC neurons also evidently miniSOG^+^ (arrowhead). Scale bars: 1, 2 µm (**b** and **c**, respectively) or 300 nm (insets). (**d,e**) On-section staining using uranyl acetate (UA) and lead citrate (Pb). Adjacent thin sections from the same specimen used in **c** were used. Note the enhanced contrast on DAB-labelled and non-DAB regions: membranes, synaptic densities (magenta arrows) and mitochondria (green stars) and cytosol. Scale bars: 300 nm (**d**,**e**). (**f**) TEM micrographs from a sample treated using ROTO, with *en bloc* UA and Pb. Highly contrasted membranes on labelled PN (using *GH146-GAL4*) is evident. Yellow dashed lines: PN neuropil boundary. Scale bar: 2 µm. TEM micrographs showing cytosol (**g**) or synapse (**h**) targeted miniSOG in PN dendrites (using *Mz19-GAL4*). Left, labelled cell body corresponds to a single adPN. Synapse labelling was achieved using Synatotagmin-miniSOG, contrasting the pre-synapse and synaptic vesicles. The presence pre-synaptic components in PN dendrites are consistent with previous observations made under light microscopy and EM^69^,^74^,^75^. The specimens were treated with ROTO (**g,h**), with *en bloc* UA and Pb (**h**). Scale bars: 5 µm and for insets: 500 nm (for **g**), 1 µm or 300 nm (mid and right insets for **h**, respectively). (**I,j**) SBEM micrographs of a specimen containing mitochondria-targeted miniSOG in PN axons. (**i**) *xy* sections of a single labelled mitochondrion are shown. Each *z*-section is spaced 50 nm apart. Synaptic features (orange and red arrowheads) are in close proximity. (**j**) Several labelled mitochondria are visible (blue arrows). Highlighted on the right are multi-planar views of a putative gap junction (orange arrowheads) next to a labelled mitochondrion. Specimen was treated with ROTO with *en bloc* UA and Pb. TEM and SBEM images were obtained from brain neuropil regions indicated (transverse sections). Scale bars: 500 nm (**i**) and insets in **j**, or 1 µm (**j**).

Having successfully introduced these probes into specific neurons by genetic targeting, we next determined whether miniSOG contrast could be detected under EM. Photo-oxidised samples were initially processed using standard osmium staining, followed by epoxy resin embedding. Ultrathin sections from brain samples that express membrane-targeted miniSOG (myr-miniSOG) in Mushroom body (MB) neurons (also known as Kenyon cells or KCs) or projection neurons (PNs) were imaged under TEM (Fig. 2b–f). Label contrast was visible in superficial (vertical lobe tip, <10 µm from brain surface) and deeper layers (peduncle, ~100 µm from surface; mid-brain) of the brain (Fig. 2b,c). Consistent with membrane localisation, the miniSOG-DAB label was localised to the membrane and proximal regions of the cytoplasmic space.

**Figure 3:**
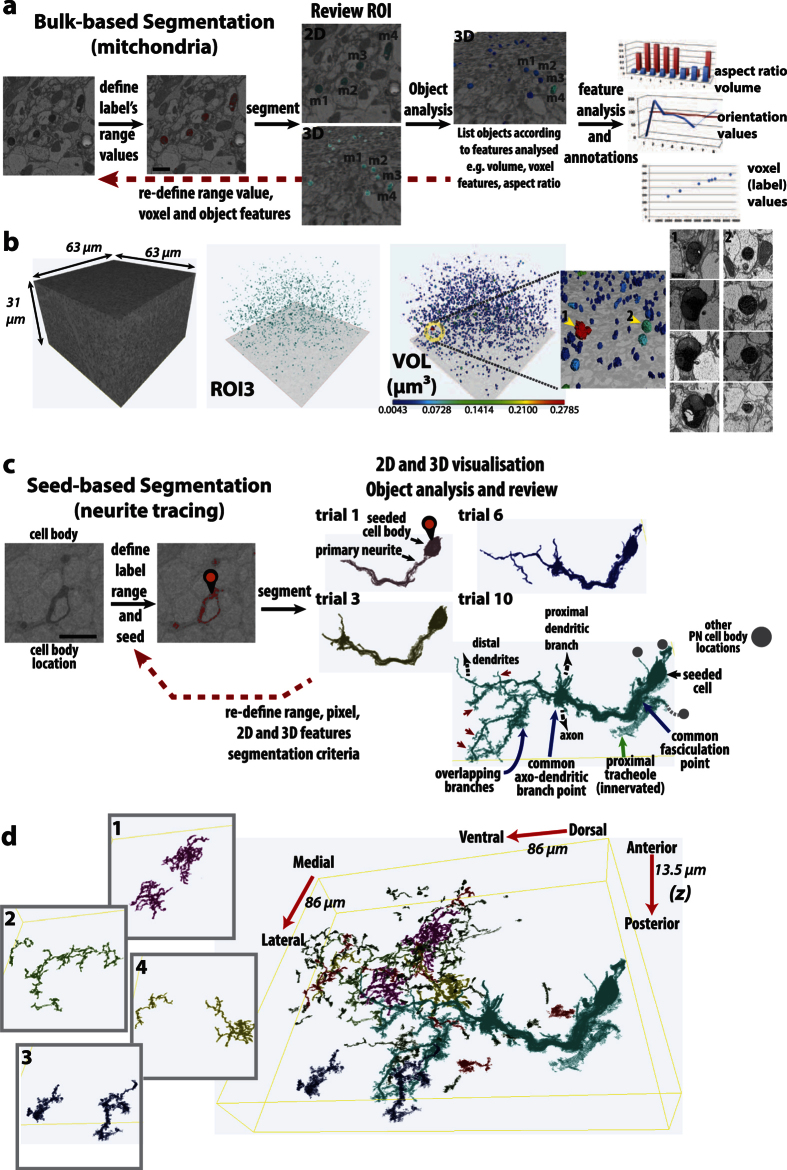
EM volume acquisition and computer-assisted segmentation of labelled volumes. (**a**) *Bulk* segmentation was applied to the mitochondria-miniSOG labelled volume. Selected range values, corresponding to miniSOG contrast (red), correlated with the labeled mitochondria. Following initial segmentation, further filtering steps were applied (see Methods). The segmented subset (ROI3) can be examined in 2D and 3D. An Object Analysis procedure produces a mesh and lists the objects identified, with their analysed features. The volume feature for a subset of mitochondria (m1–4) is displayed. The graphs on the right are for illustration purposes only. Scale bar: 1 µm. (**b**) Summary of the mitochondria-miniSOG found within the acquired volume. The desired subset (ROI3, cyan-green) can be visualized in 3D and feature-displayed according to volume (VOL); colour-indexed blue–red, from the smallest (0.0043 μm^3^) to largest (0.2785 μm^3^), respectively. The ultrastructure of the largest mitochondrion (1) can be inspected further and compared against another (2). (**c**) *Seed-*based segmentation used to trace cytosol-miniSOG labeled neurites. Following range value selection, a seeding point (pin icon) was placed at the cell body, initiating the 3D segmentation process. Several trials were performed, whereby successively lower pixel intensities were sampled (trial/ROI 1–12). Trial/ROI1 traced primary neurites of the seeded cell body. Subsequent trials incorporated increasingly distal structures that included thin protrusions from dendrite segments (red arrowheads). Other traces represent overlapping segments. These ‘crossovers’ occur when several PNs are labeled; having common cell body locations, branching points or overlapping branch segments. Given its proximity and elevated contrast, a PN-innervated trachiole (green arrow) also becomes incorporated when low range values were used. Scale bar: 500 nm. Thus, two distinct events take place as increasingly lower values are sampled. Distal branches and terminal structures are included to the primary seed. However, non-related, overlapping neurites, cell bodies and contrasted structures can also become incorporated. (**d**) By seed segmentation, additional traces were also performed within the sub-volume, based on range values in Trial10. A subset of these neurites (1–4) is highlighted, and a composite (with the labeled PN cell) is shown on the right. The different colour highlights are used to aid visualization.

**Figure 4:**
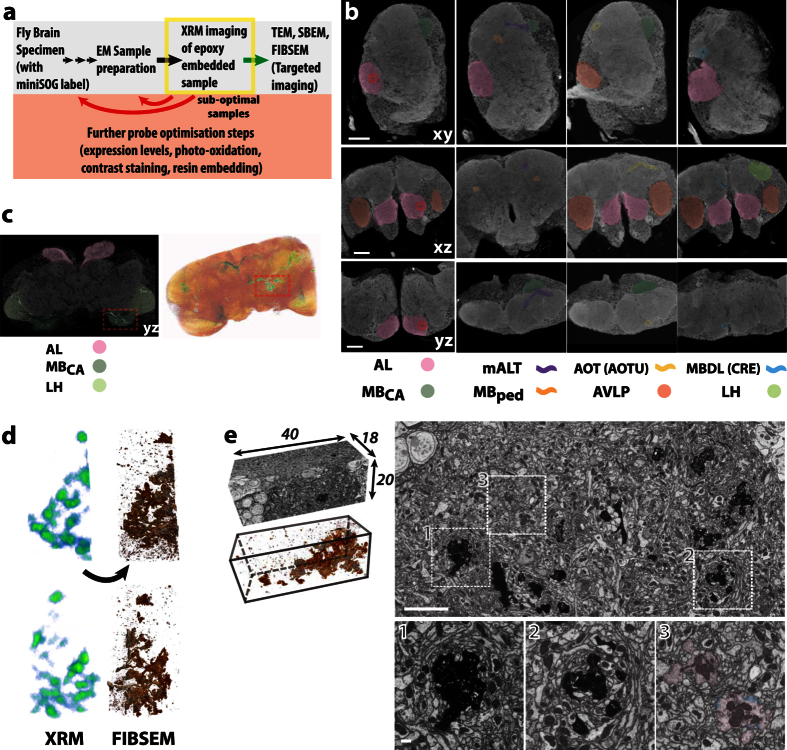
Whole fly brain miniSOG detection with correlative XRM-FIBSEM imaging. (**a**) Schematic XRM-EM workflow used to screen fly brain samples optimized for heavy metal contrast and for targeted volume acquisition. (**b**) XRM tomograms were viewed from 3 virtual plane sections: xy, xz and yz. Many notable neuropil regions were discernable, highlighting the AL, MB_CA_, and anterior ventrolateral protocerebrum (AVLP). Visible tracts included the MB_ped_, mALT the Anterior Optic tract (AOT) and the Median Bundle (MDBL). These were previously seen under light microscopy^51^. Each vertical set of 3 sections focuses on one particular region of the brain (AL, mALT, Anterior Optic Tubercle (AOTU) and Crepine (CRE),, respectively). Their slice coordinates are 603, 745, 560 and 1041 for XY; 413, 708, 470 and 434 for XZ; 903, 305, 317 and 611 for XZ. The inter-slice distance is 200 nm. Total volume dimension of the tomogram is 378 × 390 × 380 µm. Scale bars: 50 µm. (**c**) Left, A single virtual XRM section image of a fly brain specimen with cytosol-miniSOG labeled PNs. The AL, MB_CA_ and LH locations are highlighted. Right, 3D volume rendered image of the entire specimen. When threshold is applied, the miniSOG contrast is recognisable, appearing in green and red, based on a blue-red color ramp for low-high signals. Note the uneven PN labeling on the left side of the brain. As such, the right MB_CA_, boxed in red, was selected for FIBSEM volume acquisition. (**d**) Left, Correlated threshold images from XRM (green-red) and FIBSEM (as copper-silver for low-high signal intensities) tomograms, shown from two perspectives. (**e**) Left: FIBSEM volume of MB_CA_ (dimensions indicated in µm; voxel size: 10 nm isotropic). Below, thresholding reveals the miniSOG label. Right: Single *xy* section, with higher magnification from the inset regions, below. The high contrast, appearing as black, corresponds to miniSOG labeled PN terminals. A single non-miniSOG labelled PN terminal is highlighted, in pink, for comparison. Due to technical difficulties, it was not possible to obtain higher resolution of synapses from this specimen. However, some synaptic densities and vesicles are discernable, some of which are outlined in blue. This specimen was treated using ROTO only. Scale bars: 5 µm, 500 nm for inset.

Given that the miniSOG and cellular contrast using standard osmium staining appeared weak in these samples, we next explored ways to enhance the contrast in brain tissue. Differences in osmium and heavy metal staining lead to variations in the labelling of cellular and tissue structures, affecting contrast and image detection on the electron microscope used^46^,^47^,^48^,^49^. Cytoplasm, membrane boundaries, organelles and synaptic densities can be enhanced by contrast stains. While on-section treatments (uranyl acetate and Reynold’s lead citrate) for TEM is routine, this procedure is highly labile^46^. For volume imaging, this needs to be tightly controlled so that all sections achieve consistency. For fly brain samples, while on-section stains enhanced the miniSOG contrast, the use of *en bloc* (bulk) stains provided a good alternative as contrast on the miniSOG-DAB and the surrounding ultrastructure was also enhanced (Fig. 2d–f). Furthermore, *en bloc* procedures are not only applicable for block-face imaging (SBEM and FIBSEM), but they also help to reduce charging effects by grounding the sample with heavy metals^47^. Even by Reduced Osmium-Thicarbohydrazide-Osmium (ROTO) staining alone, good levels of contrast can be detected when imaged under TEM, SBEM and FIBSEM (Fig. 2g; also Figs 3 and 4).

### miniSOG contrast detection in other neuronal sub-compartments

When visualised under TEM or SBEM, we found the miniSOG probes we had designed to target to cytosol, synapses or mitochondria were also correctly localised and DAB labelling produced a good level of contrast in these subcellular compartments (Fig. 2g–j). These probes are useful in several ways. First, synapses and mitochondria are highly relevant to neuronal function and are best studied at nm-resolution: genetic labels can be used to visualise them with cell-targeted precision. Second, large numbers of labelled structures can be selectively visualised in very large volumes (see Fig. 3). Third, some probes can act as proximity markers, by labelling relevant neurons or associated structures without obstructing other relevant ultrastructures. As illustrated in Fig. 2i and j, putative chemical synapses and gap junctions can be distinguished close to mitochondria-labelled miniSOG. Last, acting as a whole cell tracer, the cytosol-targeted miniSOG has been useful in outlining labelled neurons (see Fig. 3 below).

### Enhanced contrast based segmentation from miniSOG-labelled EM volumes

We next generated EM volumes from miniSOG-labelled samples. Different acquisition and segmentation strategies were explored to see how to best visualise the label from image stacks. From the *Drosophila* brain, images were collected from the different regions of olfactory processing centres^50^, such as the lateral horn (LH, or lateral protocerebrum) that is a higher order olfactory processing centre and target site of PN axons, and the Antennal Lobe (AL), which is the olfactory information processing site between olfactory sensory axons and projection neuron dendrites. These image stacks were acquired on SBEM microscopes that have different sensitivities and configurations (see Methods). From both image volumes, robust contrast on miniSOG and the surrounding ultrastructure (membranes, organelles and synaptic compartments) was visible (Fig. 3, see Videos 2 and 3).

Given the enhanced contrast, segmenting miniSOG-DAB labelled structures should be straightforward. While manual segmentation is the most direct, we focussed on automating segmentation for several reasons. First, computational tools can readily identify labelled structures from the image stack by thresholding. This was the case when *bulk* segmentation was applied to a SBEM volume that had mitochondria-targeted miniSOG (Fig. 3a and b, Video 2). Second, once defined, such regions of interest can be further interrogated computationally for desired features of interest. For instance, we can determine pixel values, volume, aspect ratio and orientation properties of the segmented objects (Fig. 3a; Video 2). Third, we find computer-assisted visualisation is able to detect subtle differences in the contrast due to miniSOG labelling. This was particularly useful when applied to low contrast-low signal SBEM images, as shown in the seed segmentation example, thus achieving greater accuracy in neural tracing (Fig. 3c and d, Videos 3 and 4). Fourth, and related to the above, computational tools will have a robust record of the selection criteria used. This is useful if the procedure needs to be replicated across datasets, or altered to define, correct or specify for new or existing features or sub-features. Last, computational tools can readily exploit 3D segmentation tools in the first instance. This aids quicker review over the objects identified (Fig. 3).

Note however that while initial results from such segmentation strategies can be obtained relatively quickly, cell biological and neuroanatomical expertise is necessary to review the identified objects. Following initial review, iterative segmentation steps help to refine the desired objects (Fig. 3, see Discussion). As these probes can also be visualised under fluorescence microscopy, EM traces can be cross-validated with the light-based images. Cross-validation can also be performed using Micro-CT/XRM, see below.

### Micro-CT/XRM visualisation and correlation to brain regions for targeted volume EM acquisition

We were motivated to test micro-CT/XRM as an application to, 1) select optimally stained samples for EM data acquisition (prior to lengthy EM volume imaging), 2) for its ability to image whole brains non-destructively and, 3) to obtain cell biological and neuroanatomical information across very large volumes at sub-micron resolutions (Fig. 4a). Osmium-treated and epoxy-embedded fly brains were imaged on a Zeiss Xradia Versa 520 lab-based scanner. From these scans, many features were recognisable. Images were similar to those obtained from light microscopy and from scans using synchrotron-based X-ray sources^51^,^52^, respectively (Fig. 4b). In addition, our XRM images also highlighted individual cell somata from the cortical rind (perikaya) surrounding the brain, as well as axon tracts. Both appear negatively stained. Surrounding cell membranes and densely packed neuropil regions such as the antennal lobe (AL), mushroom body peduncle (MB_ped_) and calyx (MB_CA_) and optic lobe lamina and medulla layers showed higher levels of staining (appearing white or light grey in Fig. 4b, see also Video 5).

A recent study showed miniSOG generated contrast in mouse brain tissue can be detected using XRM^43^. When miniSOG labelled *Drosophila* brains were scanned, contrast from labelled PN neurons clearly highlighted axon tracts and terminal boutons (appearing white in Fig. 4c; Video 6). However, labelling from within the AL glomeruli was not seen. One possible reason for this is the high level of native contrast in this densely innervated region makes any genetically introduced contrast less likely to stand out.

As part of the EM workflow, we transferred the miniSOG labelled sample to a FIBSEM microscope. From the initial XRM scan, we noticed that the bilateral miniSOG^+^ PN axonal structures were unevenly labelled within the specimen, with the left side having lower signal at the PN terminals in the MB_CA_ (Fig. 4c). The reason for this is unclear, it may have occurred during the photo-oxidation or the post-staining process. Nonetheless, the XRM scan allows us to select the right MB_CA_ as the ideal region of interest from which to acquire a FIB image sub-volume, which was approximately 0.075% of the total brain volume.

We established a correlation between FIBSEM and XRM by registering the SEM field image on the sample surface to virtual slices from the XRM volume, enabling the precise targeting to the region of interest. Using FIBSEM, we acquired image stacks from the mushroom body calyx (Ref volume: *150915-R2/5*; volume dimensions: 40 × 20 × 18 µm; voxel dimension: approx. 10 × 10 × 10 nm; z-slices: 1800). Here we find the overall contrast and of the level of miniSOG labelling of the cytosol of PN axon terminals were very high. Comparisons between the XRM and FIBSEM tomograms showed the label contrast closely matched each other (Fig. 4d; Video 7). We are interested in the Mushroom Body calyx (MB_CA_) as it is a higher order olfactory processing region where PN axon terminals synapse with Kenyon cell (KC) dendrites^50^. Indeed, closer inspection of the FIBSEM images revealed very fine diameter KC dendrites surrounding the PN boutons (Fig. 4e), similar to previous reports^53^,^54^.

In summary, using XRM, we were able to screen candidate samples for optimal labelling characteristics and perform site-specific volume image acquisitions using FIBSEM. Having whole brain XRM images also provides many cell and neuroanatomical relevant features that are useful for subsequent reconstruction and analysis.

## Discussion

Probe labelling is a key method in biological imaging. The ability to highlight specified structures helps decipher how macromolecular complexes, cellular components, cells and tissues are organized, giving insight into their function. In neuroscience, given the diversity of molecular, cellular and physiological substrates, fluorescent probes have been used extensively in cell biological, neuroanatomical and neurophysiological studies^55^,^56^,^57^. Yet adopting them for the purpose of light-based neural circuit reconstruction has limitations as it is unclear how segmentation can be implemented on densely labelled neurites across large dimensions and at high resolution. Volume EM techniques are therefore favoured, with acquisition volumes of >6,000,000 μm^3^ and 4–50 nm voxel resolution already reported^5^. However, given the large amount of high-density data, working out how individual axon, dendrites and synapses are represented as neural network-based entities requires substantial reconstruction time^2^.

In this study, we have focused on EM-visible probes, which are genetically-encoded, highly compatible with sample preparation methods and capable of high resolution visualisation. These criteria facilitate the ease of labelling, promote ultrastructure preservation and contrast generation. This allows for a single EM modality to detect the genetically introduced label and discriminate cellular ultrastructure and both are acquired at identical nm-scale resolutions. Our feasibility study acquired volume datasets from olfactory centres in the *Drosophila* adult brain that are densely innervated. From these studies, several observations were made, which are discussed below.

When expressed in *Drosophila* neurons, we find that miniSOG works effectively when targeted to distinct cellular sub-compartments. This is a marked improvement over other EM probes such as HRP, which have limited targeting capabilities^32^,^33^,^39^, or ReASH, which necessitates cell-toxic conditions for probe introduction^58^. One initial concern was whether the inaccessibility of blue light in deeper tissue regions might hamper the labelling process^34^. With protocols used here, DAB label was visible in the mid-brain, ∼100 µm depth. One protocol adaptation was to perform photo-oxidation using blue-light LEDs (see Methods).

One area of continued development will be to gauge how much DAB label is sufficient for image detection. This is delicate balance, avoiding under- and over-labelling the sample, and multiple parameters, such as probe expression level, localised targeting, labelling density, DAB photo-oxidation, fixation conditions, heavy metal staining and microscope settings play a role in the EM visualisation of the labelled product. Thus, our current approach is based on multiple trials, where many samples are collected over the course of the DAB photo-oxidation step. The tandemisation approach, by attaching multiple miniSOG probes to a single targeting motif, together with multi-copy transgene expression, helps not only to promote DAB labelling, but also provides a broader array of labelled samples, increasing the chance of finding the optimal specimen. While this does not impact on the study’s conclusions, ideal probes ought to be small and minimally expressed in order to reduce the possibility of disrupting cell function. Related to this, our fluorescence imaging tests suggest that not all probes tested are currently optimal. For example, while the mitochondrial-targeted miniSOG does not perturb PN axon or dendrite targeting, as judged by light microscopy, mitochondria labelled miniSOG appeared aggregated at the cell body and less frequently in distal axon termini. Closer EM-level examination shows some mitochondria appear smaller and fragmented. Axon fragmentation was also prevalent in the LH in the specimen of a 7-day old adult fly. This suggests high levels of mitochondria-miniSOG expression may lead to neurotoxicity over time. Perhaps related to the above, we find expressing high-levels of mitochondria resident proteins, whether untagged or tagged with miniSOG, also resulted in similar aggregation, loss (or partial loss) of neurons and neurite segment phenotypes (unpublished observations). In *C.elegans,* high levels of miniSOG expression in the mitochondria also caused neurotoxicity and cell death^59^. Nonetheless, given that label contrast can be detected in mitochondria and serve an important purpose, refined probes will facilitate further improved use. Further improvements will also come from re-engineered probes^60^,^61^ and by tuning their expression close to wild-type levels, while providing sufficient levels of discrimination under EM and XRM.

To aid segmentation, labelling probes should fulfil several key criteria. Exhibiting highly localised contrast is critical. The label needs to be detected uniformly throughout the regions and structure of interest targeted. Label generation needs to be minimally disruptive for image detection; any background artefact introduced has to be minimal. Several aspects were addressed in this study. Highly localized miniSOG contrast was consistently observed. Segmentation throughout the volume suggests artefacts and background effect were minimal. In any imaging paradigm, there are always concerns regarding the extent of targeting and labelling density any probe can achieve. In the course of neurite tracing using cytosolic miniSOG, we found that segmentation errors can result from signal drop-off, particularly at dendritic branch points and at large axonal segments where it was more difficult to achieve high or uniform levels of labelling. Such errors are easily detected when imaging at nanometre-scale resolutions. Nonetheless, with expert knowledge of projection patterns and by looking at the underlying ultrastructure, such errors can be easily rectified (see Video 8 for examples). Thus, one area of continued development will be to determine how each designed probe can achieve optimal labelling at targeted sites of interest. One easy way is to allow for more DAB label to develop. However, overdevelopment can generate labelling artefacts and result in the loss of resolution (Supplementary Figure 2). Masking, where the underlying ultrastructure is obscured by DAB labelling, is also another concern.

Labelling approaches provide a simple way to extract information from complex biological images. As we have shown using miniSOG, labelled neuronal components can be easily segmented from high-density EM images. Computational tools work well in this respect as discrete pixel-based criteria can be applied to the entire volume. As low- and high-order features become attributed to each segmented object, this forms a valuable way of linking them within the volume, facilitating large-scale, comparative and interpretive analysis. Future work will explore how such tools work best and the types of information that can be obtained with each probe. More tools will inevitably be needed to complement and complete the reconstruction process. This might include semi-automated and rules-based segmentation algorithms that reflect neuronal morphologies and cellular structures. While this manuscript was being finalised, we were encouraged to find similar approaches were also being developed to exploit genetically encoded EM marker based neural tracing in mouse neurons^35^. Similar approaches to adopt unsupervised segmentation of labelled neurons will be useful in our future work.

One interesting property of computer-assisted segmentation is the high degree of morphological complexity found in the dendritic arbours of labelled PNs, particularly in the lateral regions (Example 3 in Fig. 4d). The organisational significance of these intricate branch protrusions is unclear. Interestingly, high-resolution analysis in the AL of silkmoth *Bombyx mori* also show similar dense protrusions from the dendritic shafts of PNs^62^. These features are also reminiscent of the ‘twig-like’ or ‘spine-like’ structures recently found throughout *Drosophila* neurons analysed, where thin actin-rich and microtubule-free terminal neurites show high levels of synaptic input^63^. Future work will determine whether these tufted protrusions on PN dendrites carry specific molecular and regional identities or synaptic patterns required for AL connectivity.

Many aspects of fixation, labelling and contrast generation are not well understood and have led researchers to reinvestigate EM sample preparation methods to achieve better consistency^47^,^48^,^49^,^64^,^65^. The inability to predict if EM specimens are optimal leads to wasted efforts, which on any given microscope incurs days-weeks of acquisition time. If sub-optimal image volumes become incorporated into the segmentation workflow, this adds to further waste. This prompted us to investigate XRM as a method for checking sample quality. Our initial testing has shown it to be critical prior to EM volume acquisition. In addition, XRM has become an invaluable imaging tool, revealing cell body and long-range projections patterns as well as discrete brain regions. Future work will explore how the XRM can be used as a part of a reconstruction workflow to trace neurons across scales of whole brains, brain regions, axon and dendrite projections and ultimately leading to synaptic connectivity patterns under EM.

In summary, our study explores the implementation of EM-based reporters to label neurons in a whole brain setting. We propose a set of tools that uses genetically-encoded probes, enhanced contrast staining, automated image volume acquisition and computer-assisted segmentation for the dissection of complex neural circuits. By combining genetic labelling with the structural analysis of molecularly defined components, such targeted approaches help uncover local network motifs and synaptic connectivity patterns. They are especially valuable in identifying targeted neurons even within a restricted EM volume (which can be acquired in shorter periods of days) that does not include enough of the neuronal arbour for cell type identification by morphology. With these approaches, we can contemplate scaling towards comparative analyses, to determine how circuit properties are altered under the influence of genetic, environmental and physiological cues. With enough examples from different samples and brain regions, their insights can contribute to wider EM neural reconstruction efforts that are taking place across larger regions and to the general understanding of the nervous system organization.

Although our own research focuses on neural circuitry, these tools will also be very useful in cell and developmental biology and disease model studies. With a focus on large dimension, high-resolution based imaging, EM probes can be used to identify rare features (e.g. transient structures or cell types that arise from morphogenetic or pathological events), or to acquire large datasets for ultrastructural studies of specific cellular components.

## Methods

### *Drosophila* stocks and immunohistochemistry

Brain samples were dissected from 3–7 day old adult males. GAL4 drivers used have been previously described^66^,^67^,^68^. Immunostaining was performed as previously described^69^. miniSOG antibodies were generated in guinea pigs (Eurogentec S.A., Seraing, Belgium).

### miniSOG design, vector construction and Drosophila transgenesis

miniSOG DNA was custom synthesised (GeneArt, Life Technologies) for optimal codon expression in *Drosophila*, with the addition of restriction sites at the 5′ and 3′ ends to facilitate in-frame fusion cloning. To make tandem versions, several fragments were mixed together during restriction cloning into *Drosophila* expression vectors^70^. Probes were targeted to the plasma membrane using a Src myristoylation tag^70^. miniSOG fusion to the *Drosophila* Eb1 protein resulted in cytoplasm localisation. *Eb1* DNA (*Eb1-PE*; Flybase ID: FBpp0089088) was made synthetically, as above. Synaptic targeting was achieved by fusing miniSOG to Synaptotagmin, similar to previous strategies^69^. Mitochondria-localisation was based on miniSOG fusion to a *Drosophila* gene product (CG18769; Isoform PG; Flybase ID: FBpp0300165), based on its sequence homology to the Mitochondria Calcium Uniporter gene (MCU). Its labelling characteristics in *Drosophila* neurons (Figs 2i and 3b) are very similar to previous studies^34^. All miniSOG fusions were incorporated at the COOH-terminal to the above-targeted proteins. *Drosophila* transgenic lines were derived from germline transformations (Bestgene Inc., CA, USA) using the ΦC31/attP system^71^.

### miniSOG expression in S2 cells and *Drosophila* brain

miniSOG was initially tested in *Drosophila* S2 cells as described previously^72^ and analysed under confocal microscopy. miniSOG probes were expressed *in vivo* using neuronal GAL4 driver lines to express UAS-miniSOG products in mushroom body Kenyon cells (OK107-GAL4^66^) or projection neurons (PNs) [using the GH146-GAL4^68^ [or Mz19-GAL4, that label PN subsets innervating glomerulus DA1, VA1d and DC3)]^67^. Fluorescence images for miniSOG, tagRFP and mKate2 fluorescence data were acquired as previously described^36^ (http://evrogen.com/spectra-viewer/viewer.shtml).

### Sample preparation for miniSOG photo-oxidation

Brain samples were processed similarly to previous studies^36^, with minor modifications. *Drosophila* adult whole brains were fixed overnight in 4% paraformaldehyde, 0.5% glutaraldehyde in 2 mM CaCl_2_, 0.1 M Cacodylate, (pH 7.4). These samples were kept cold whenever possible. Fixed brains were rinsed in chilled cacodylate buffer and were incubated in blocking buffers (in 0.1 M cacodylate) sequentially for 10–15 minutes each time in (1) 10 mM Glycine, (2) 10 mM Potassium Cyanide, (3) 5 mM 3-aminotriazole and (4) 5 mM Mersalyl acid, to reduce non-specific DAB staining. miniSOG photo-oxidations were initially performed using a 150W Xenon light source (Lambda DG-4alpha, Sutter Instruments, CA, USA) with a 20×/NA 0.75 Plan Fluor Nikon objective that was mounted on a Nikon TE2000 microscope. At 470 nm, this was approximately 1 W/cm^2^ with 3.8 mm^2^ field of view. Subsequently, a LED-based photo-oxidation unit was built (CREE; Code: 2216679; Part: XBDROY-00-0000-000000L01). This achieved illumination stability and several samples could be processed simultaneously. Samples were photo-oxidised for 2–20 minutes, being kept at 4 °C throughout. For some probes, it was possible to monitor the progress of this process due to the presence of the brownish DAB precipitation. This was not the case for mitochondria-localised miniSOGs.

### Brain sample preparations for osmification, resin embedding and image acquisition

Different osmium and heavy metal stains were applied, following previous protocols^46^,^48^. The variations in osmification included the use of 1% osmium tetraoxide (standard), 1.5% potassium ferricyanide with 1% osmium tetraoxide (reduced osmium or RO) or reduced osmium-thiocarbohydrazide-osmium (ROTO; RO as above, followed by 1% thiocarbohydrazide and a second round of 1% standard osmium). For contrast stains, *en bloc* uranyl acetate and lead aspartate were applied to whole brain samples. On-grid staining included uranyl acetate and Reynold’s lead citrate; applied for 2–5 minutes prior to TEM imaging^46^. Epoxy (Durcupan ACM, Sigma Aldrich #44611) embedded samples were sectioned and visualised by TEM (Technai T12 Spirit, FEI, Hillsborough, OR, USA), using a 2 K × 2 K camera detector (BM-Orius, Gatan, Pleasanton, CA, USA). Following TEM verification, miniSOG-positive specimens were imaged using SBEM or FIBSEM. SBEM data was acquired using a 3View serial-block-face imaging unit (Gatan, Pleasanton, CA, USA) installed either with a high-vacuum Merlin or Sigma variable pressure (VP) field emission-SEM (Carl Zeiss Microscopy, Jena, Germany), equipped with either a 32 K × 32 K or a 8 K × 8 K camera detector, respectively. We noticed that SBEM operating at high vacuum will produce better contrast and resolved images than those operating under variable pressure mode. This is a direct result of the absence of gas scattering and higher surface sensitivity, enabling lower beam energies to acquire data during probe scanning. Similar results in brain tissue were recently reported elsewhere^19^. Apart from SBEM, volume data was also collected on an Auriga Crossbeam FIBSEM, equipped with the ATLAS 5 scan generator and software tools (Carl Zeiss Microscopy, Jena, Germany).

### Image processing, visualization and segmentation analysis

Confocal microscopy data were acquired and processed as previously^69^. For EM images, individual TEM micrographs, single or sub-stacks of SBEM and FIBSEM images were inspected using Fiji^73^. Image pre-processing was applied as necessary to remove background noise, improve visualization and figure presentation. These filters included Thresholding, Smoothing, Gaussian blur, Non-Local Means and Sharpening. Image stack alignments were also performed as necessary using StackReg on Fiji. Figures are presented in 8-bit to reduce file size.

Large EM volume stacks were visualized and analysed using ORS Visual SI Advanced software (Object Research Systems Inc., Montreal, Canada; Carl Zeiss Microscopy GmbH, Jena, Germany). To facilitate better performance on the workstation, image datasets were downsampled by binning (3 × 3 × 1).

Following SBEM volume acquisition, two computer-assisted segmentation approaches were tried. For the mitochondria-miniSOG labelled volume (Ref: *140807-R04;* 63 × 63 × 31 µm; voxel dimension: 6.3 × 6.3 × 50 nm; number of z-image sections: 624), given that the label was well contrasted and segregated, *bulk* segmentation was applied. Range values as determined from pixel probe readings and interactively setting minima and maxima threshold values. Once the selected range correlated with the labeled structure, a Region Of Interest (ROI) can then be defined. However, initially, all pixels corresponding to the desired range will be segmented from the image volume. High contrast but low volume objects were eliminated from the segmented dataset as these were corresponded to non-specific background signals (cutoff: <10 voxels; <0.0002 µm^3^). A further filter based on the variance intensity, eliminated larger, cell-based structures, such as highly contrasted cell membrane segments. The segmented objects were then examined in 2D and 3D, comparing them against the underlying ultrastructure. Following this, an Object Analysis function was applied. This produced an object mesh and listed all the segmented objects identified, together with their analysed features (voxel intensities, volume and orientation properties). SBEM image volume was acquired, using a 3View-Merlin SEM, from a specimen (*Sample 10*) that has been treated with ROTO with UA and Pb stains.

A *seed-*based segmentation method was applied to cytosol-miniSOG labelled PNs as that the label appeared contiguous and thus likely to have overlapping voxels (see Video 3). After defining the range values, a seed point (black pin icon) was placed at the cell body location. A Point and Click seeding function initiates the 3D segmentation process. Several trials were performed based on defined range intensities. Set to common maximum, increasingly lower range intensities were sampled (trial/ROI 1–12). Each step change represented 0.2–0.4% of the true range. This SBEM volume (Ref volume: *141127-R01*; volume dimension: 86 × 86 × 13.5 µm; voxel dimension: 10.5 × 10.5 × 25 nm; z-sections: 541) was acquired using the 3View-Sigma VP SEM, from a ROTO treated specimen (*E1*). It was also used in TEM (Fig. 2g). The volume stack corresponds to mid - posterior location on the right AL. For segmentation analysis, the stack was image pre-processed by applying non-local means (sigma = 5).

Video editing was performed using ORS Visual SI Advanced and on iMovie (Apple Inc., Cupertino, CA, USA).

### XRM/micro-CT analysis of epoxy embedded Drosophila brains

X-ray microscopy (XRM) was performed using a Zeiss Xradia Versa 520 (Carl Zeiss X-ray Microscopy, Pleasanton, CA, USA). The following settings were used to collect tomograms. The emission source was set at 70–80 kV and 6–7 W. The samples were placed 8.9 mm from RA-Source and 6.4 mm for RA-Detector. A LE1 source filter was used. A 40× optical magnification objective was used, resulting in a field image of 0.409 mm × 0.409 mm. With a 1950 × 1950 pixel image size, this translates to a 200 nm voxel size. The estimated reconstructed spatial resolution is 700 nm. The total scan time lasted 22–24 hours, based approximately on 17 sec exposure time and 360° degree rotation and step size of 0.11.

## Additional Information

**How to cite this article:** Ng, J. *et al*. Genetically targeted 3D visualisation of *Drosophila* neurons under Electron Microscopy and X-Ray Microscopy using miniSOG. *Sci. Rep.* **6**, 38863; doi: 10.1038/srep38863 (2016).

**Publisher's note:** Springer Nature remains neutral with regard to jurisdictional claims in published maps and institutional affiliations.

## Supplementary Material

Supplementary Video 1

Supplementary Video 2

Supplementary Video 3

Supplementary Video 4

Supplementary Video 5

Supplementary Video 6

Supplementary Video 7

Supplementary Video 8

Supplementary Information and Figures
