# Supplementary material for: Genetically targeted 3D visualisation of Drosophila neurons under Electron Microscopy and X-Ray Microscopy using miniSOG
Source: Sci Rep. 2016 Dec 13;6:38863. doi: 10.1038/srep38863 (PMC5153665; doi:10.1038/srep38863)
Supplement: Supplementary Information and Figures [file srep38863-s9.pdf]

Genetically targeted 3D visualisation of *Drosophila* neurons under Electron Microscopy and X-Ray Microscopy using miniSOG

Julian Ng[1, 4], Alyssa Browning[2], Lorenz Lechner[2], Masako Terada[2], Gillian Howard[3] and Gregory S. X. E. Jefferis[1, 4]

[1] Department of Zoology, Downing Street, Cambridge, CB2 3EJ, United Kingdom
[2] Carl Zeiss X-ray Microscopy Inc., 4385 Hopyard Rd., Suite 100, Pleasanton, CA 94588, USA
[3] Cell Biology Division, MRC Laboratory of Molecular Biology, Francis Crick Avenue, CB2 0QH, United Kingdom
[4] Neurobiology Division, MRC Laboratory of Molecular Biology, Francis Crick Avenue, CB2 0QH, United Kingdom

Supplementary Information

Supplementary Figure 1: Effect of increasing miniSOG motifs on DAB labelling

- A-B. Transmitted light images of *Drosophila* brains post-DAB labelling. The photo-oxidation process can be monitored by the presence of DAB precipitate, which is seen in MB neurons, where membrane-miniSOG is expressed. The DAB labelling extended over a 5-20 minute period. This process is blue light dependent as samples kept in the dark or exposed to green light do not show these stains (not shown). Blue light exposure of control fly brain samples (w^{1118}) also did not result in such patterns (not shown). Expressing multiple copies as tandem motifs (A) or multi-copy transgenes (B) further enhanced DAB labelling.

Video 1: Volume rendered images of mitochondrial- targeted miniSOG from confocal microscopy

Two video sequences showing fluorescent image stacks acquired from PN neurons expressing mitochondria-miniSOG (magenta, by immunostaining) together with control myr-GFP (green), acquired at AL or MB_{CA} and LH locations, respectively, as shown in Figure 1I. Following a 2D section-section review, 3D volume renders were generated from the stacks. Thresholded GFP signals were indexed as a colour ramp (blue - red for low - high signal intensities). The mitochondria-miniSOG labelling is also color-indexed as dark brown-silver for low-high signal intensities. As shown in the first video sequence, other non-PN neurons are also

present in the Mz19-GAL4 expression pattern, anterio-dorsal to the AL. To visualise mitochondria-miniSOG localised to PN neurons, a mask (dark purple) was generated from the AL-specific, GFP-positive traced region and applied to the mitochondria-miniSOG image stack. Note that a large fraction of labelled mitochondria aggregated at the cell somata.

Supplementary Figure 2: Effect of DAB over-development *in situ*

- TEM micrographs, sectioned at the AL, showing synaptic vesicle labelled miniSOG in PN dendrites (expressed using *Mz19-GAL4*). From a larger panel, three higher magnification images were taken and shown on the right. Note the effect of excessive photo-oxidation/DAB development not only results in higher contrast but also in localised artefacts in adjacent structures such as in mitochondria (highlighted in pink). These show the appearance of holes (blue) and tiny tears (yellow arrows). These effect on mitochondria may be due to free radical damage generated from photo-oxidation. Tears and holes could result from reduced resin infiltration and subsequent fragmentation during sectioning, hence the highly electro-lucent areas. This specimen (*S1-4*) was treated with the ROTO only. The image corresponded to the anterior of the AL. Scale bars: 5 micron or 500nm (insets), as indicated in panel.

Video 2: Segmentations based on bulk thresholding of mitochondria labelled miniSOG

This video accompanies Figure 3A-B. The first sequence illustrates the segmented mitochondria (ROI3, cyan-green) obtained from the EM volume, viewed across a 2D stack sequence. In the second clip, the volume is rendered in 3D showing the segmented mitochondria. A 3D mesh is created from the segmented objects, where individual structures are displayed according to size; blue – green - red; smallest (0.0043 μm^3) – largest (0.2785 μm^3). The meshed 3D structure can be compared section-section in 2D against the ultrastructure (inset video). For example, the large red mitochondrion (**1**, in Figure 3B) that appear to be breaking down, as internal cristae no longer exist and miniSOG labels appear aggregated. For comparison, a second smaller mitochondrion is shown (**2** in Figure 3B).

Video 3: Seed-based segmentation of a single cytosol-miniSOG labelled PN cell

This video accompanies Figure 3C and initially shows a partial stack of the EM volume labelled with cytosol-miniSOG (*141127-R01*, surface 1-180 sections out of the total of 541; volume: 85×85×4.5 micron). These were image-processed by thresholding and Gaussian blur (sigma=3) and two rounds of Sharpen using Fiji. Note the miniSOG contrast on a whole PN cell body and on many labeled neurites that appear contiguous throughout subsections of the AL. Other partially imaged labeled cell bodies are also present in the volume, at the surface and to the right side of the stack. In the thresholding stage, each intended ROI are indicated by the highlighted range values (red). 12 trials were then performed,

sampling pixels at increasing lower intensities but set to a common maximum value. Note the increasing length and complexity of 3D structures segmented for each ROI.

Video 4: Further identification of cytosol-miniSOG labelled neurites within volume

This clip shows some of the other neurite traces found within the image volume, as described in Figure 3D.

Video 5: XRM image scans of wholemount fly brain

As described in Figure 4B, some of the cell and neuroanatomical features seen in the XRM scans on a whole fly brain, shown from different planes.

Video 6: XRM scans of whole fly brain containing cytosol-miniSOG labelled PNs

This clip shows miniSOG labelled PNs seen in the XRM scans in the fly brain, and accompanies Figure 4C.

Video 7: XRM-FIBSEM acquisition workflow

This clip illustrates the correlative XRM-FIBSEM workflow, which uses non-destructive XRM to screen miniSOG labeled PN neurons in a whole fly brain, followed by FIBSEM volume acquisition, as described in Figure 4.

Video 8: Examples of over-segmentation errors in cytosol-miniSOG labelled PNs within the AL

These two video sequences illustrate some examples where it was not possible to fully segment neurites due to signal drop-off. In the first clip, this occurs as pixel intensities were either very low or had labelling discontinuities along the dendritic segments. This was frequently observed in distal or very thin branch points. In some cases, such discontinuities are considered minor and so merge functions can be easily performed, as illustrated. The second clip shows labelled PN axons in the posterior half of the AL (Ref: *150105-R2*; volume: 51.4×51.4×5 micron; voxel: 6.3×6.3×25nm; Sample-*E1*; ROTO only). Here over-segmentation occurs due to the incomplete labelling pattern of cytosol-miniSOG in wide axonal segments. In both cases, having both cell biological and neuroanatomical expertise and the underlying ultrastructure helps to resolve these errors.

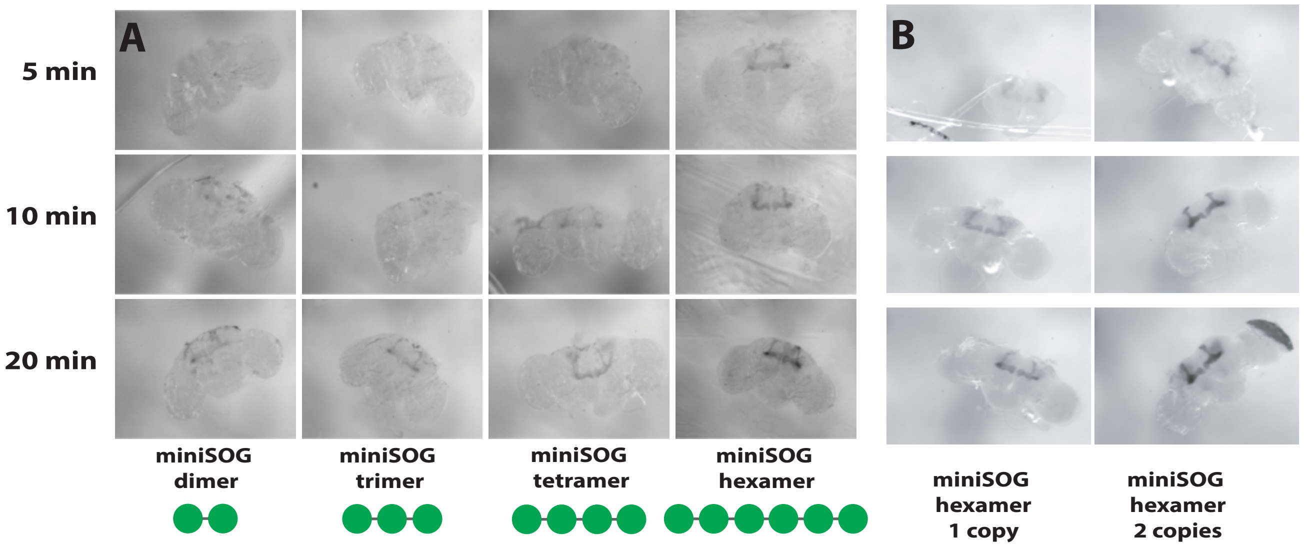

Supplementary Figure 1
Ng

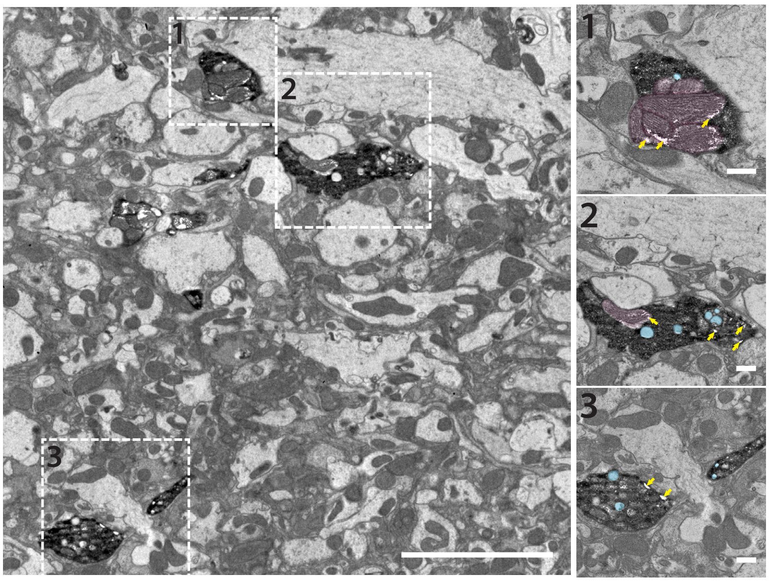

Supplementary Figure 2 Ng
